# Lipid-enriched and protein-enriched enteral nutrition limits inflammation in a human endotoxemia model

**DOI:** 10.1186/cc9803

**Published:** 2011-03-11

**Authors:** M Kox, T Lubbers, JJ De Haan, JW Greve, JC Pompe, BP Ramakers, P Pickkers, WA Buurman

**Affiliations:** 1 Radboud University Nijmegen Medical Centre, Nijmegen, the Netherlands; 2 Maastricht University Medical Centre, Maastricht, the Netherlands; 3 Atrium Medical Center, Heerlen, the Netherlands

## Introduction

Enteral administration of lipid-enriched nutrition was previously shown to attenuate inflammation and organ damage via a cholecystokinin-mediated vagovagal reflex in animal studies. The current proof-of-principle study investigates the immunomodulatory potential of enteral lipid-enriched and protein-enriched nutrition during experimental human endotoxemia.

## Methods

After an overnight fast, 18 healthy male subjects received an intravenous bolus of *Escherichia coli *lipopolysaccharide (LPS; 2 ng/kg). Subjects in the fasted group (*n *= 6) were deprived of food throughout the study, while subjects in the intervention groups were fed either enriched (*n *= 6) or isocaloric control nutrition (*n *= 6) via a nasojejunal tube, starting 1 hour prior to LPS administration until 6 hours afterwards.

## Results

**Figure 1:**
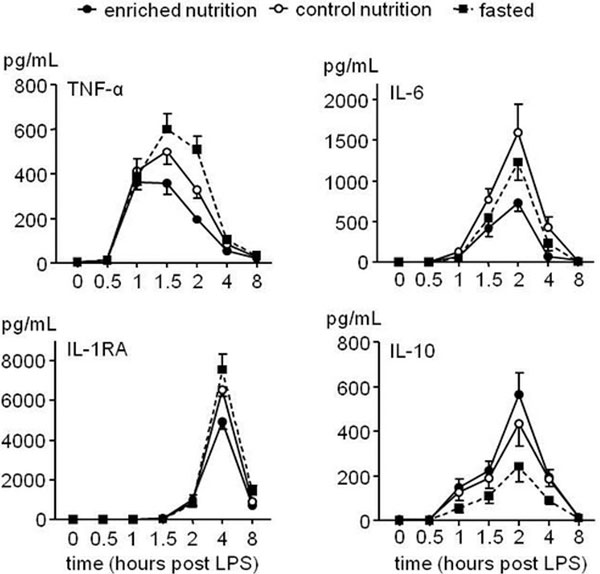
**Plasma cytokine levels in the three experimental groups**.

LPS administration resulted in a marked inflammatory response. Continuous postpyloric administration of nutrition increased plasma cholecystokinin levels. Enriched nutrition attenuated circulating levels of the proinflammatory cytokines TNFα and IL-6 and the IL-1 receptor antagonist compared with control nutrition (all: *P *< 0.01) and fasted subjects (all: *P *< 0.05). Additionally, enriched nutrition augmented the anti-inflammatory response, reflected by increased IL-10 release compared with fasted subjects (*P *< 0.0001). See Figure 1.

## Conclusions

The current study establishes the anti-inflammatory potential of enriched nutrition in humans. The immediate anti-inflammatory effect of enriched nutrition suggests that the beneficial effects are mediated via a cholecystokinin-dependent vagovagal reflex. Enteral administration of enriched nutrition is a promising intervention to modulate the immune response in the early course of systemic inflammation.

